# Recent Advances in the Design, Modeling, and Control of Flexure-Based Nanopositioning Stages

**DOI:** 10.3390/mi16121312

**Published:** 2025-11-23

**Authors:** Yijie Liu

**Affiliations:** 1 Coal Mining Research Institute, China Coal Technology and Engineering Group Co., Ltd., Beijing 100013, China; yj-liu19@tsinghua.org.cn; 2 CCTEG Intelligent Strata Control Technology (Tianjin) Co., Ltd., Tianjin 300392, China; 3 State Key Laboratory of Intelligent Mining and Strata Control, Beijing 100013, China; 4 State Key Laboratory of Tribology in Advanced Equipment, Department of Mechanical Engineering, Tsinghua University, Beijing 100084, China; 5 Beijing Key Laboratory of Transformative High-End Manufacturing Equipment and Technology, Department of Mechanical Engineering, Tsinghua University, Beijing 100084, China

**Keywords:** flexure, nano-positioning, compliant mechanism, dynamics modeling, motion control

## Abstract

Flexure-based nanopositioning stages have emerged as indispensable tools in advanced fields such as nanotechnology, semiconductor manufacturing, and biomedical engineering, where nanometer-scale precision is paramount. This paper presents a comprehensive review of the state-of-the-art in flexure-based nanopositioning, systematically examining the three critical and interconnected domains of geometric design, theoretical modeling, and advanced control strategies. This review begins by analyzing fundamental design principles, including motion decoupling, stiffness-range trade-offs, and various structural topologies (serial, parallel, and hybrid), highlighting how they achieve high precision and reject disturbances. It then delves into analytical and computational modeling techniques, from pseudo-rigid-body models and beam theory to finite element analysis, which are essential for predicting system behavior and guiding design optimization. A core section of this review is dedicated to control methodologies, providing a critical analysis of active resonant control for damping mechanical vibrations, classical and robust control for stability under uncertainties, and modern adaptive and learning-based techniques for handling nonlinearities and time-varying dynamics. Furthermore, this review addresses persistent challenges such as bandwidth limitations, performance trade-offs, and the integration of complex multi-axis systems. Finally, it outlines future research directions, emphasizing the promising potential of data-driven modeling, artificial intelligence-enhanced control, and a holistic mechatronic co-design approach to push the boundaries of precision, speed, and robustness in next-generation nanopositioning systems. This work aims to serve as a systematic reference and synthesis for researchers by integrating a vast body of literature and providing a clear perspective on the development of high-performance nanopositioning stages.

## 1. Introduction

The relentless pursuit of miniaturization and precision in modern science and engineering has transformed nanopositioning systems from specialized laboratory tools into essential technologies across diverse industries [1,2,3]. These systems, capable of controlling motion with nanometer-scale resolution, underpin advanced manufacturing, precision metrology, and emerging nanotechnologies [4,5,6]. In the semiconductor industry, nanopositioning stages are indispensable for photolithography, wafer inspection, and mask alignment, where sub-nanometer accuracy is required for fabricating integrated circuits with ever-decreasing feature sizes [5,7]. In precision metrology, flexure-guided stages enable ultra-accurate measurements in scanning interferometry and surface analysis, facilitating material and device characterization at the atomic scale [8,9,10]. Similarly, life science applications such as super-resolution microscopy and single-cell manipulation rely on nanopositioning stages for achieving and maintaining nanometric accuracy, while dual-stage actuators in hard disk drives employ nanopositioning for high-density data storage.

Flexure mechanisms guide motion through the elastic deformation of compliant elements, eliminating mechanical contact and thereby achieving frictionless, backlash-free movement [11,12,13,14]. The absence of sliding interfaces minimizes wear and hysteresis, resulting in exceptionally smooth, deterministic, and repeatable motion. Moreover, the high stiffness of flexures enhances vibration resistance and mechanical bandwidth, while their monolithic structure offers excellent long-term stability [15,16,17,18]. Consequently, flexure-guided nanopositioning stages have become the preferred solution in a range of high-precision applications—from atomic force microscopy (AFM) scanners achieving sub-nanometer probe positioning [19,20,21] to semiconductor wafer alignment systems requiring nanometric registration accuracy [12]. The adoption of flexure technology has been a key enabler of progress in micro- and nanoscale manufacturing, measurement, and manipulation [22,23,24].

The performance of flexure-based nanopositioning stages is commonly characterized by four interrelated metrics: range of motion, precision, bandwidth, and stability [8,25,26]. The achievable range is typically limited by material strain to the micrometer-to-millimeter scale, but innovations such as compound flexure amplifiers and optimized compliant mechanisms have extended this into the centimeter range without compromising accuracy [5,27]. Flexure systems excel in precision—modern stages routinely achieve nanometer or sub-nanometer resolution [19]. High stiffness-to-mass ratios confer elevated resonant frequencies, often in the tens to hundreds of hertz, enabling rapid scanning and effective disturbance rejection [5,28]. Their monolithic structure further ensures robustness against vibration, thermal drift, and varying payloads [12].

A central challenge in nanopositioning stage design lies in balancing these interdependent performance metrics. Increasing travel range generally reduces stiffness and bandwidth, while enhancing dynamic response may limit achievable range or load capacity. Overcoming such trade-offs necessitates careful design optimization, including strategies like passive stiffness compensation and hybrid actuation [29]. Recent research trends emphasize multi-objective optimization and co-design approaches that jointly consider mechanical structure and control system design, enabling better trade-off management among range, precision, and speed [8,25]. This integrated perspective is increasingly recognized as essential for advancing the state of the art in nanopositioning performance.

**Figure 1:**
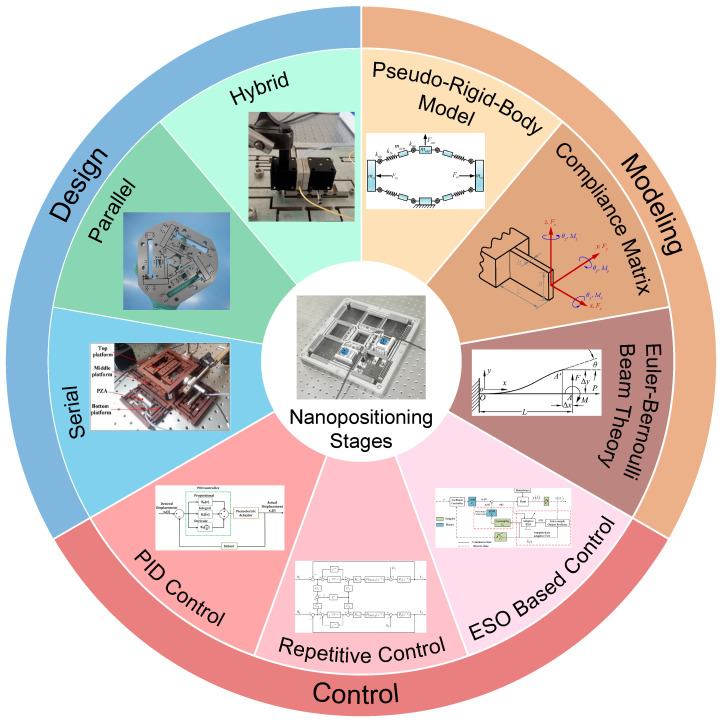
Overview of the flexure-based nanopositioning stages [30,31,32,33,34,35,36,37].

To provide a comprehensive perspective, the remainder of this review is organized as follows. Section 2 examines the geometric design of flexure-based nanopositioning platforms, covering fundamental design principles, common flexure topologies (serial, parallel, and hybrid configurations), and advanced design methodologies such as displacement amplification, multi-DOF decoupling, and topology optimization. Section 3 discusses modeling and analysis techniques for flexure-guided systems, including analytical models (e.g., pseudo-rigid-body and stiffness matrix methods) and high-fidelity numerical simulations that capture their nonlinear and dynamic behavior. Section 4 reviews control strategies for nanopositioning stages, ranging from classical PID feedback control and cross-coupling compensation to modern robust, adaptive, and feedforward approaches aimed at maximizing precision and bandwidth. Section 5 presents the major challenges facing current flexure nanopositioning stages—such as range versus precision trade-offs, hysteresis and creep nonlinearity, and control of multi-axis dynamics—and offers a future outlook on emerging solutions (integrated design, smart materials, improved algorithms, etc.). Figure 1 provides a schematic overview of the key concepts and scope covered in these sections. Finally, Section 6 provides concluding remarks and suggestions for future work, summarizing key insights from this review and highlighting opportunities for further advancement in precision mechatronic systems.

## 2. Geometric Design of Flexure-Based Stages

The geometric design of flexure-based nanopositioning stages is a critical determinant of their ultimate performance, dictating key operational characteristics such as travel range, precision, bandwidth, and cross-coupling between axes [38,39,40]. Unlike conventional mechanisms that rely on sliding or rolling contacts, flexure mechanisms achieve motion through the elastic deformation of their constituent material. This frictionless, monolithic motion guidance eliminates mechanical contact issues like friction, backlash, and stiction, enabling ultra-high precision and repeatability at the nanometer scale. However, this reliance on material elasticity introduces a unique set of design challenges, including managing parasitic motions, balancing stiffness versus range, and ensuring long-term fatigue life. The design process involves a complex interplay of materials science, mechanical structure, and even control strategy to create a flexure system that is both kinematically and dynamically optimized for its intended application. This section provides a comprehensive review of the fundamental principles, structural topologies, and advanced methodologies that govern the geometric design of these sophisticated motion systems.

### 2.1. Fundamental Design Principles and Challenges

The design of flexure-based nanopositioning stages is governed by core principles aimed at maximizing performance while mitigating inherent limitations. The primary goal is to achieve the desired motion in one or more degrees of freedom (DOFs) while minimizing unwanted parasitic errors in other directions [12,28,41,42,43]. This requires careful consideration of the mechanism’s kinematics, stiffness characteristics, and stress distribution. There are inherent trade-offs between key performance metrics – for example, obtaining a large range of motion typically comes at the expense of stiffness and vice versa [44,45,46,47]. A long travel range often demands more compliant structures, which can lower natural frequencies and reduce disturbance rejection capability. Conversely, maximizing stiffness improves bandwidth and load capacity but limits elastic deformation and thus range. Furthermore, the elastic nature of flexures means that stress concentrations are a major concern, as they can lead to material fatigue and, ultimately, failure of the mechanism [48,49,50]. Addressing these challenges is paramount for developing stages that are not only precise but also robust and reliable over extended operational periods. The following subsections explore these fundamental principles and challenges in greater detail, providing a foundation for understanding the more advanced design concepts discussed later in this review.

#### 2.1.1. Motion Decoupling and Parasitic Error Minimization

A central challenge in the design of multi-axis flexure stages is achieving kinematic decoupling, where motion along one axis does not induce unwanted motion (parasitic error) in another [51,52,53]. This is particularly critical in high-precision applications like AFM and semiconductor lithography, where even nanometer-level cross-talk can compromise performance. Parasitic errors can manifest as slight translational or rotational motions in constrained degrees of freedom and are often exacerbated by the inherent compliance of flexure mechanisms. In multi-DOF systems, maintaining motion purity also requires adequate out-of-plane stiffness to resist external disturbances and minimize off-axis deformations. Designers employ various strategies to minimize these effects, notably through symmetric architectures and redundant constraints [54]. For example, a mirror-symmetric arrangement of flexure modules can balance internal forces and moments, while adding redundant constraint beams increases off-axis stiffness, improving the disturbance rejection capability of the mechanism [54]. In practice, symmetric parallelogram flexures combined with supplemental constraint beams have been shown to substantially reduce parasitic motions in planar stages with multi-millimeter workspaces. Such geometric layouts ensure that any deformation inducing an undesired motion is counteracted by a symmetric counterpart, resulting in near-cancellation of parasitic shifts.

The effectiveness of these design strategies is often quantified by measuring the cross-talk between axes. For example, in a modified double-parallelogram flexure (DPF) stage designed for a 50 mm travel range, the static cross-talk was found to be less than 2.9%, indicating highly effective decoupling [5]. This improvement was achieved by shifting the problematic resonant modes of the original stage from approximately 25 Hz to above 86 Hz in the modified design, without significantly increasing the footprint or compromising the primary motion. In other words, dynamic parasitic modes were pushed well outside the operating bandwidth of the system through geometric reconfiguration. These results demonstrate that dynamic performance is intrinsically linked to geometry and its ability to manage parasitic effects. The use of finite element analysis (FEA) is indispensable in this process, allowing designers to predict and visualize mode shapes associated with parasitic motions and to iteratively refine the geometry so that these modes lie beyond the system’s working frequency range. The ultimate goal is a mechanism in which the primary motion modes are well-separated in frequency from any parasitic modes, simplifying control design and enabling higher-speed operation. Nevertheless, minor residual couplings often remain and may need to be handled via advanced control techniques. Contemporary flexure stages that integrate both careful decoupling design and active compensation can achieve cross-axis errors under 0.5% and sub-nanometer precision in practice.

#### 2.1.2. Stiffness and Range Trade-Offs

One of the most fundamental trade-offs in flexure mechanism design is between stiffness and range of motion [19,28,55,56]. High stiffness is desirable because it raises the system’s natural frequencies (allowing a higher control bandwidth) and improves resistance to external disturbances and vibration, as well as load capacity. However, making a flexure stiffer (for instance, by using thicker or shorter flexure elements) reduces its ability to deform, thus limiting travel range. Conversely, a design optimized for a large range of motion will inherently be more compliant, leading to lower resonant frequencies and reduced disturbance rejection. This trade-off is a central theme in long-range nanopositioning stage design, where the challenge is to achieve millimeter-level travel while maintaining nanometer-level precision.

Designers navigate this trade-off using a combination of strategies. One approach is to incorporate displacement amplification mechanisms, such as levers or bridge-type flexure amplifiers, to magnify the output motion of a high-stiffness, short-stroke actuator like a piezoelectric stack. For example, multi-level lever mechanisms (e.g., Scott-Russell linkages and bridge amplifiers) have been used in mirror-symmetric flexure stages to achieve amplification ratios above 10, extending the range of piezo stacks to hundreds of microns [57]. In one design, a piezo-driven 5-DOF stage used an integrated half-bridge lever with spring leaf flexures to achieve a Z-axis travel of about 100 µm, far greater than the actuator’s free stroke [58]. Another common strategy is to employ different types of actuators for coarse and fine motion. For instance, long-stroke voice coil motors (VCMs) can provide millimeter-scale motion which is then nested with high-precision, short-stroke piezoelectric actuators for fine positioning in a dual-stage configuration [59,60,61]. Such hybrid actuation allows the system to cover a large overall range without sacrificing nanoscale precision. A novel stage uses a normal-stressed electromagnetic actuator with an integrated negative-stiffness element, achieving a tunable stiffness ratio of 3.24. This enables in-situ adjustment of the stage’s dynamic performance as required [62].

The choice of flexure geometry also plays a crucial role in balancing stiffness and range. Beam flexures can be shaped in Z, Π, L, T or other configurations to tailor the compliance in desired directions while maintaining support in others [57,58,63,64,65,66,67]. For instance, Z-shaped and Π-shaped flexures (as mentioned earlier) offer a favorable mix of lateral stiffness and axial range, making them popular in long-travel stage designs. Optimizing the geometric parameters of flexure elements is often a multi-objective problem—increasing thickness or reducing length improves stiffness and dynamics but cuts into range, and vice versa [25,68]. Accordingly, designers frequently perform multi-objective optimization (e.g., Pareto optimization) to identify designs that best satisfy conflicting requirements. Computational design frameworks have been developed to automate this process, using FEA-based surrogate models or parametric studies to find an optimal compromise between range and stiffness. Such methods have yielded flexure stages that approach the theoretical limits of the stiffness–range trade-off, providing large travel (several millimeters) while retaining high resonant frequencies and resolution.

#### 2.1.3. Stress Concentration and Fatigue Life

A critical consideration in the design of flexure mechanisms is the management of stress concentrations and the prediction of fatigue life [69,70]. Flexure hinges are deliberately made to be the most compliant parts of the mechanism, which also makes them the most highly stressed under deflection [48,49,50]. The repeated elastic deformation of these hinges during operation can lead to the initiation and propagation of microscopic cracks, a phenomenon known as fatigue. If left unchecked, fatigue can ultimately cause catastrophic failure of the flexure, rendering the entire nanopositioning system inoperable. A key objective in design is therefore to ensure that stress levels in flexure hinges remain well below the material’s fatigue limit for the expected number of cycles. This requires careful analysis of stress distributions using tools such as FEA, and often the incorporation of features that mitigate stress concentrations. Sharp corners and sudden changes in cross-section, for example, should be avoided or relieved with fillets to reduce peak stresses [49,69]. Studies have shown that adding generous radii at hinge corners can dramatically lower the stress concentration factor and improve fatigue life. In one analytical and experimental investigation, a corner-fillet flexure hinge design achieved significantly larger elastic deflection before fatigue onset compared to a traditional straight-notch hinge, confirming that fillet radii effectively extend hinge lifespan.

Beyond modifying hinge geometry, designers may employ alternative flexure layouts or materials to alleviate stress hotspots. For instance, using distributed compliance (long, thin flexural segments) instead of concentrated short hinges spreads out the bending strain, thereby lowering peak stress at any single point [71]. An example is a compliant guiding mechanism with orthogonally oriented flexures, which was shown to reduce maximum stress and improve fatigue resistance by over threefold relative to a conventional design [50]. Selecting fatigue-resistant materials (e.g., precipitation-hardened spring alloys) and limiting the operational strain range are also important considerations. Overall, a combination of geometric refinement (fillets, compliant segments) and informed material choice is used to ensure flexure hinges can endure the billions of cycles often required in nanopositioning applications.

### 2.2. Structural Topologies for Multi-DOF Systems

The choice of structural topology is a primary decision in the design of multi-DOF flexure-based nanopositioning stages, as it fundamentally defines the mechanism’s kinematic and dynamic behavior. The arrangement of flexure joints and linkages dictates how motions in different axes are generated and coupled, as well as the overall stiffness distribution and modal characteristics of the stage. Broadly speaking, flexure stages can be categorized as serial kinematic, parallel kinematic, or hybrid combinations of the two. Each topology offers distinct advantages and drawbacks in terms of simplicity, range, precision, and dynamic performance [72]. This section reviews these topology types and their implications for nanopositioning stage design.

#### 2.2.1. Serial Kinematic Structures

Serial kinematic structures are a common and straightforward approach to creating multi-DOF motion systems [59,71,73,74,75]. In a serial configuration, each DOF is provided by a separate flexure module or stage, and these modules are stacked in series (cascaded). For example, an XYZ nanopositioning stage might be constructed by mounting an X-axis flexure stage, a Y-axis stage on top of the X, and a Z-axis stage on top of the Y. This approach offers a modular design that is relatively easy to conceptualize and implement. Because each axis is driven and guided independently, decoupling in the control sense can be simpler, and the travel range in each axis can be maximized by dedicating an actuator to it. Serial designs have been widely used in commercial nanopositioning stages and microscope sample scanners due to their simplicity and modularity.

**Figure 2:**
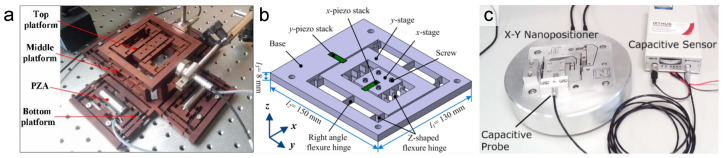
Serial kinematic structures. (**a**) A 6-DOF compliant stage based on bridge-type amplifier [30]. (**b**) A 2-DOF piezoelectric-driven stage [76]. (**c**) A 2-axis serial-kinematic nanopositioning stage [77].

Figure 2 presents a series of nanopositioning stages based on a serial kinematic architecture. In this design strategy, motion axes are stacked sequentially, which simplifies the mechanical structure and control design by physically decoupling degrees of freedom. The depicted examples are frequently employed in applications requiring multi-axis coordination over a large range, such as high-speed AFM and nano-visualization systems. While this stacking can lead to a cumulative reduction in the resonant frequency of sequentially stacked axes, it offers a straightforward approach to achieving complex motion patterns.

However, serial stacking of flexure stages also has notable disadvantages. The moving mass seen by the lower-axis actuators increases with each additional stage stacked on top, which can lower the resonant frequencies and bandwidth of the system. For instance, a compact long-range stage that linked an X motion stage in series with a Y-$\theta_{z}$ stage achieved a large travel (hundreds of microns) but required careful dynamic design to mitigate the mass loading effects of the stacked assembly [74]. Moreover, any small parasitic rotation or tilt in a lower stage will carry into the stages above it, potentially compounding positioning errors. The cumulative tolerances of each module can also lead to a slight loss of orthogonality between axes. In practice, high-performance serial-kinematic flexure systems often include compensation mechanisms or control calibration to counteract these error accumulations. Despite these challenges, serial configurations remain popular for their conceptual simplicity and because they allow each axis to be optimized somewhat independently.

#### 2.2.2. Parallel Kinematic Structures

Parallel kinematic structures represent a more advanced, high-performance alternative to serial configurations for multi-DOF nanopositioning stages [42,45,61,69,73,78,79,80,81]. In a parallel mechanism, all actuators are connected to a single moving stage through multiple flexure linkages, and motions in different DOFs are achieved simultaneously through the coordinated deformation of these linkages [82,83]. This topology inherently provides a high degree of geometric coupling and constraint, which can be leveraged to achieve excellent motion decoupling and stiffness. An example is the parallel XY stage in which four symmetrically arranged flexure modules (often in a double parallelogram configuration) support a central moving stage [28]. Parallel-kinematic flexures avoid the issue of accumulated mass and error from stacking, since each actuator effectively works in unison on the same stage. As a result, parallel stages can attain higher resonant frequencies and more isotropic behavior. For instance, a flexure parallel mechanism was demonstrated to achieve a 10 mm × 10 mm travel range with only 0.1–0.3% cross-axis coupling by virtue of its symmetric parallel constraint design [84]. Similarly, a 6-DOF parallel flexure stage achieved sub-nanometer resolution and sub-arcsecond rotational precision while maintaining a first resonant frequency around 396 Hz due to its highly symmetric and stiff parallel architecture [79].

**Figure 3:**
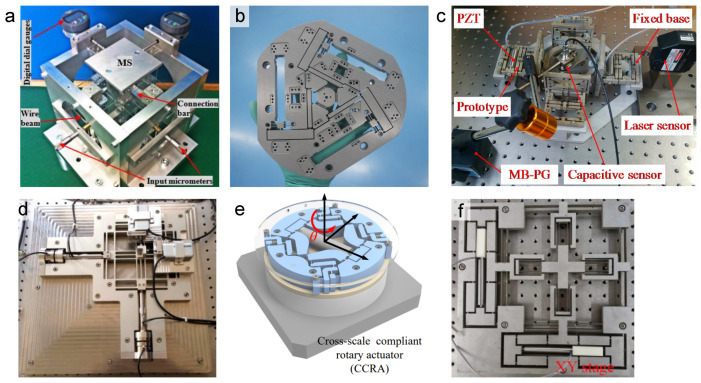
Parallel kinematic structures. (**a**) A prototype of XYZ compliant parallel mechanism [85]. (**b**) A spatial 6-RRRR compliant parallel nanopositioning stage [31]. (**c**) A piezoelectric-actuated micro-/nano-compliant stage [33]. (**d**) A 2-DOF Compliant Parallel Mechanism [86]. (**e**) A cross-scale compliant rotary actuator [87]. (**f**) An XY piezo-actuated compliant micro-positioning stage [34].

Figure 3 illustrates a collection of representative parallel kinematic structures developed for flexure-based nanopositioning. These architectures leverage symmetrically arranged limbs to guide the motion of a central stage, a design principle that often yields high stiffness, high resonant frequencies, and improved dynamic performance. The selected examples, spanning designs from purely translational XY stages to planar systems with three degrees of freedom, demonstrate the versatility of the parallel kinematic approach in achieving objectives such as motion decoupling, reduced parasitic errors, and enlarged travel ranges.

Despite their performance benefits, parallel kinematic flexure designs are generally more complex to analyze and design. The kinematics and load distribution in a parallel system are coupled and must be solved simultaneously. Additionally, parallel flexures can suffer from internal stress coupling—when multiple actuators drive the stage, internal constraint forces can arise if the flexures are not perfectly arranged. Careful design (e.g., exact constraint design) is required to avoid over-constraining the mechanism. Manufacturing parallel flexure systems can also be challenging, as they often involve intricate monolithic cuts or assembly of multiple flexure components with tight tolerances. Nonetheless, numerous state-of-the-art nanopositioning stages employ parallel topology to achieve exceptional decoupling and dynamic performance. For example, a parallel XY compliant manipulator was developed to support electron-beam lithography, achieving a 1 mm × 1 mm planar travel with only 0.5% cross-coupling error and a 56 Hz first mode by optimizing a parallel leaf-spring linkage design [55]. Parallel kinematic flexures thus excel in applications demanding high agility and precision, albeit with increased design complexity.

#### 2.2.3. Hybrid Serial–Parallel Configurations

Hybrid serial–parallel configurations combine elements of both serial and parallel topologies in an attempt to leverage the advantages of each while mitigating their respective disadvantages [44,73,88]. In a hybrid approach, certain axes or degrees of freedom may be realized in parallel, whereas others are added in series. A serial-kinematic tri-axial AFM scanner combined normal-stressed electromagnetic actuators for the X-Y plane with a piezoelectric Z actuator. The design achieved a 212.7 µm X-range at a 1313 Hz resonance and demonstrated both large-area coarse scanning and high-speed fine scanning in AFM imaging tests [89]. Another example is a tip/tilt stage mounted on top of a parallel XY stage – the in-plane motions use a parallel flexure mechanism for decoupling and stiffness, while the out-of-plane rotations (tip/tilt) are achieved by a serial addition on top [39]. This arrangement isolates the delicate parallel mechanism from having to support large rotations, and the rotational module can be optimized separately. Another example of hybrid design is the integration of a coarse positioning stage with a fine parallel flexure stage [45]. For instance, a large-stroke XY table driven by lead-screws or voice coils can carry on its moving platen a smaller high-speed parallel flexure stage for nanometric fine positioning. Such a coarse-fine hybrid allows millimeters of travel range along with nanometer resolution.

**Figure 4:**
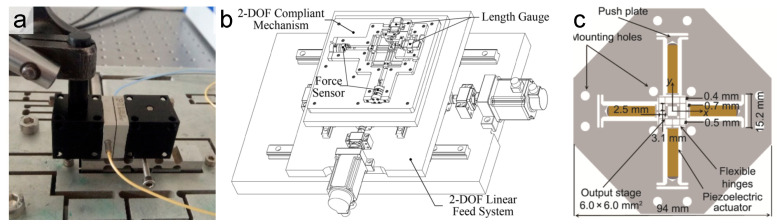
Hybrid serial–parallel configurations. (**a**) A 3-DOF motion device based on the flexible mechanism [32]. (**b**) A macro-micro-manipulator [90]. (**c**) A high-speed nano-positioning stage [91].

Figure 4 illustrates a class of hybrid serial–parallel kinematic configurations, which aim to synergize the distinct advantages of both archetypes. These designs strategically combine parallel modules for their high stiffness and motion decoupling capabilities with serial stacks to achieve multi-axis motion or facilitate actuator isolation. This approach provides a versatile framework for addressing complex design challenges, such as managing actuator saturation, expanding the overall workspace, and optimizing the dynamic performance.

The key benefit of hybrid configurations is that they offer a compromise between the simplicity of serial designs and the performance of parallel designs [73,88]. Each portion of the hybrid can be tailored to its role: the parallel sub-mechanism handles precision motion and decoupling in critical DOFs, while the serial sub-mechanism provides extended range or additional DOFs that would be impractical to realize in parallel. Research prototypes of parallel-serial integrated stages have demonstrated, for example, an XY motion with a compact parallel flexure guiding the motion, combined with a long-stroke Z actuator in series for extended vertical range [58]. Another design employed a 3-PPP parallel flexure for XYZ motion with a separate tip-tilt module mounted on top for angular alignment, effectively achieving a 5-DOF system with minimal cross-coupling [43]. The challenges in hybrid systems lie in managing the interface and dynamics between the serial and parallel components. Care must be taken so that the added serial elements do not reintroduce significant compliance or error that undermines the parallel mechanism’s benefits. With thoughtful engineering, hybrid topologies can offer a versatile solution when neither pure serial nor pure parallel designs alone meet all the requirements.

#### 2.2.4. Topology Optimization for Performance Enhancement

Topology optimization is a computational design method that optimizes the material layout within a given design space for specified loads, boundary conditions, and performance objectives [8,25,50,60,68,71,72,80,92,93,94,95]. In the context of flexure mechanism design, topology optimization can be used to discover non-intuitive flexure geometries that achieve superior performance by distributing material in an optimal way. This is especially powerful for complex multi-DOF stages or when seeking extreme performance characteristics (e.g., maximum stiffness-to-mass ratio or tailored compliance in specific directions). For example, an optimization algorithm might remove material selectively to lighten a flexure mechanism while ensuring that required stiffness and resonant frequency targets are met. The resulting design often has an organic, latticed appearance that would be difficult to conceive through manual design.

In recent years, several researchers have applied topology optimization and other computational techniques to flexure stage design [41]. One study demonstrated a flexure-based XY positioning stage optimized via an FEA-based response surface methodology, achieving improved dynamic performance and minimal cross-coupling by iteratively adjusting cutout shapes in the flexure hinge regions [95]. The advent of additive manufacturing has further broadened the scope for topology-optimized flexures, since the often intricate geometries resulting from optimization can now be fabricated. For instance, a 3-DOF spatial flexure mechanism was topologically optimized and then 3D-printed in Ti-6Al-4V, yielding a manipulator with a 4 mm linear stroke and ±6° angular motion while maintaining good decoupling and a 119 Hz first mode [96]. These examples highlight that topology optimization can systematically push flexure designs toward the theoretical limits of performance. It should be noted, however, that the optimized designs must still satisfy practical constraints such as manufacturability and material behavior (including avoiding stress hotspots that could reduce fatigue life). Therefore, better results are obtained when topology optimization is used in tandem with engineering judgment and validation via FEA and experiments.

#### 2.2.5. Integrated Mechatronic Co-Design for System-Level Performance

The conventional sequential design process—where the mechanical structure is finalized prior to control system design—inherently limits the exploration of synergistic solutions that transcend disciplinary boundaries. In contrast, mechatronic co-designtreats the mechanical plant, actuator dynamics, and control algorithm as a unified optimization problem. This paradigm is crucial for nanopositioning systems, where the physical design dictates the dynamic model, and the model’s accuracy fundamentally constrains the achievable control performance. Co-design methodologies explicitly address this interplay, leading to holistic optimizations that are unattainable through sequential approaches.

The critical role of modeling accuracy in control performanceis a primary driver for co-design. A control law designed on an idealized, decoupled model will underperform or destabilize a physical system with unmodeled high-frequency modes or complex cross-couplings. For instance, the effectiveness of advanced control techniques is highly sensitive to the accuracy of the identified resonant frequencies and mode shapes. Inaccurate model identification can render a well-designed damping controller ineffective or even introduce instability, creating a direct feedback loop from modeling fidelity to control robustness. This interdependence necessitates a co-design approach where model identification and controller synthesis are iterative and linked processes.

Conversely, mechanical design can be strategically leveraged to simplify control challenges and enhance robustness. A prominent example is the use of mechanical design to linearize system dynamics. In the design of a large-stroke nanopositioner driven by a nonlinear reluctance actuator, researchers intentionally incorporated a compliant compensation mechanism. This mechanism counteracted the actuator’s inherent nonlinear force-displacement characteristic, resulting in a more linear relationship between input current and output force [29]. This mechanical linearizationsignificantly reduced the burden on the control system, which no longer needed to compensate for strong nonlinearities, thereby enabling simpler, more robust, and higher-bandwidth control. This case exemplifies how a deliberate mechanical design choice, guided by control objectives, can profoundly facilitate control robustness.

The implementation of co-design is increasingly enabled by computational frameworks that integrate high-fidelity, multi-physics simulations (e.g., FEA for structural dynamics) with control optimization algorithms. These tools allow for the simultaneous optimization of geometric parameters (e.g., flexure dimensions, actuator placement) and control parameters (e.g., PID gains, filter coefficients) against system-level objectives such as closed-loop bandwidth and tracking accuracy. By concurrently optimizing across disciplines, co-design uncovers solutions that balance trade-offs more effectively than sequential methods, pushing the performance boundaries of speed, precision, and robustness. As such, co-design represents the forefront of holistic system-level optimization, essential for meeting the escalating demands of next-generation nanopositioning applications.

### 2.3. Material Selection and Manufacturing Considerations

The choice of material and manufacturing process are critical aspects of flexure stage design, as they directly impact performance, cost, and reliability. The ideal material for a flexure mechanism should have a high yield strength (to allow a large elastic range), a high Young’s modulus (for stiffness), and good fatigue resistance for long operational life. Common materials include aluminum alloys, stainless steels, and beryllium copper. Aluminum alloys (e.g., 7075-T6) are lightweight and easy to machine, but have lower yield strength and fatigue endurance than steel or BeCu [65]. Stainless steel offers a good balance of strength, stiffness, and corrosion resistance, making it a popular choice for many precision applications. Beryllium copper provides excellent fatigue resistance and high strength, ideal for mechanisms requiring an extremely long lifespan and reliable spring properties. Material selection can sometimes be application-specific; for instance, titanium alloys may be used for their non-magnetic properties or Invar for low thermal expansion in specialized environments.

The manufacturing process is equally important. Traditional machining methods (milling, turning, grinding) can produce flexure mechanisms, but complex designs can be time-consuming and expensive to fabricate by subtractive means. Wire electrical discharge machining (WEDM) has become a preferred method for producing monolithic flexures, as it allows intricate 2D profiles to be cut from sheet or plate with very high precision and minimal induced stress [58,66,97]. Using WEDM, researchers have fabricated flexure stages with complex geometries that closely match the design intent and FEA models. For example, the monolithic centimeter-range XY stage mentioned earlier was cut via WEDM from a single aluminum billet, achieving the designed shape and performance without assembly errors [98]. In general, a monolithic construction (all flexures in one piece) is favored for highest precision and repeatability, whereas an assembled construction (multiple flexure components bolted or bonded) offers more flexibility in fabrication and material choice. Each approach has its pros and cons, as discussed next.

#### 2.3.1. Monolithic vs. Assembled Structures

The decision to use a monolithic or an assembled structure is a fundamental one in flexure-based stage design, with significant implications for performance and manufacturability. A monolithic structure is machined from a single piece of material, resulting in a mechanism with no joints or interfaces between components [53,65,81,98,99,100]. This inherently eliminates assembly misalignments and removes sources of backlash, friction, and wear, thereby enabling extremely high precision and repeatability. Monolithic flexure stages are typically stiffer and have higher resonant frequencies than an equivalently sized assembled stage, since there are no compliant bolted joints to add compliance or damping. Additionally, fabrication of a monolithic design can be straightforward for planar geometries—e.g., using WEDM to cut out the flexure profile from sheet stock. Many of the highest-precision nanopositioning stages are monolithic.

However, monolithic structures also have drawbacks. The manufacturing process (often WEDM or precision computer numerical control machining) can be expensive and time-consuming for complex 3D geometries. Any fabrication error ruins the entire part, which can drive up costs, especially for large or intricate stages. Moreover, monolithic designs limit the designer’s ability to mix and match materials—every section of the stage must be the same material, which might be a compromise if one desires, say, steel flexure hinges for high strength but a lighter aluminum moving stage. By contrast, an assembled structure is made of multiple components joined together (by screws, pins, bonding, etc.) [27,59,63,101,102]. This approach offers greater flexibility: different parts can use different materials optimized for their function, and simpler manufacturing processes can be used on each part before assembly. Assembled designs can also be easier to repair or adjust; individual components can be tuned or replaced without remaking the entire system.

The trade-off is that assembled structures introduce interfaces that can degrade precision. Joints may bring slight friction or free play (if not carefully preloaded), and the process of alignment during assembly can introduce small errors. For example, a piezo-driven inchworm rotary actuator was developed using multiple assembled flexure components (clamping blocks, stator, etc.), which provided flexibility in design and very high torque output, but required meticulous assembly to ensure each flexure piece was aligned for synchronous motion [103]. Generally, the overall stiffness of an assembled system will be limited by the stiffness of its joints; fastening elements must be designed to minimize compliance. Despite these issues, assembled flexures are advantageous for large-scale systems or those where modularity is needed. Indeed, some large-range industrial nanopositioning stages use an assembled frame and flexure modules to reduce manufacturing difficulty and cost, accepting a slight performance penalty in return.

#### 2.3.2. Additive Manufacturing and Its Impact on Design

Additive manufacturing (AM), also known as 3D printing, is a transformative technology that is beginning to impact the design and fabrication of flexure-based stages [81,96]. Unlike subtractive methods that carve material away, AM builds parts layer by layer from a digital model, enabling shapes and internal structures that would be difficult or impossible to create conventionally. This capability is particularly well-suited for producing topology-optimized flexure mechanisms, which often feature intricate, organic geometries as a result of optimization algorithms. Using AM, designers have fabricated flexures with complex lattice or compliant cellular structures that yield high stiffness-to-weight ratios and more uniform stress distributions than traditional designs. For example, a Ti-6Al-4V compliant parallel mechanism optimized via beam-based topology synthesis was 3D-printed using electron beam melting (EBM), achieving a multi-DOF motion with a large workspace (4 mm translation, $6^{\circ }$ rotation) while maintaining a fast dynamic response (119 Hz) and minimal cross-coupling. This would have been exceedingly difficult to manufacture without AM [96].

Beyond enabling topology optimization, AM allows for the creation of integrated, multi-material structures. It is possible, for instance, to print a flexure mechanism where the main body is a stiff metal and the flexure hinges are made from a more compliant or damping material, by using multi-material printing processes. This opens the door to flexure designs with built-in damping or vibration attenuation, something traditionally added through external means. AM also greatly accelerates the prototyping cycle: new flexure concepts can be printed and tested in a matter of days, facilitating rapid iteration in the design process. Researchers have leveraged this to experiment with novel compliant mechanisms that might have been too risky or time-consuming to machine in the past.

However, AM comes with its own set of challenges for flexure fabrication. The surface finish of printed parts is generally rougher than that of precision-machined parts, which can introduce micro-notch effects and reduce fatigue life if not post-processed. The material properties of AM parts can be anisotropic (direction-dependent) and may exhibit internal porosities or residual stresses, all of which must be accounted for in the design. Careful calibration (such as the use of an “effective thickness” correction in lattice structures) is often required to ensure the printed flexure’s performance matches the model. Despite these issues, the benefits of AM for flexure design are significant. We are likely to see increasing adoption of AM for specialized flexure stages, especially as printing technologies improve. Already, complex compliant devices—from millimeter-scale parallel mechanisms to reconfigurable antennas enabled by printed compliant hinges—have been demonstrated, showing that AM can produce reliable flexures for high-precision applications. In the coming generation of nanopositioning systems, additive manufacturing is expected to play an important role in realizing designs that were previously only theoretical, truly expanding the design space for flexure-based stages.

Table 1 provides a systematic comparison of the experimentally characterized performance metrics for representative large-stroke XY flexure stages, highlighting the common trade-offs and advancements within this field. The collated data reveals that while individual designs often excel in specific parameters—such as maximizing stroke, minimizing cross-axis coupling, or achieving high resonant frequencies—a consistent challenge lies in optimizing all performance metrics simultaneously without compromising the structural compactness. Notably, critical data like parasitic rotation and trajectory tracking error are frequently unreported, indicating a area for more standardized characterization. The comparison underscores the ongoing pursuit of designs that successfully balance nanometer-level precision over multi-millimeter travels with high bandwidth and a minimal footprint, outlining a clear trajectory for future research in high-performance flexure mechanism design.

The sophisticated designs discussed herein necessitate equally sophisticated modeling techniques to predict their performance accurately. The transition from conceptual design to a functional prototype is guided by theoretical models that bridge the gap between geometry and real-world behavior.

## 3. Modeling and Analysis of Flexure-Based Nanopositioning Stages

Accurate theoretical modeling is fundamental for the design and control of flexure-based nanopositioning stages, as it establishes the mathematical relationship between applied loads and resulting motions [107,108,109,110,111,112]. These models are typically built upon the fundamental assumptions of slender beams and small deformations, which form the basis for linear elastic theory and enable the derivation of tractable analytical solutions.Under these premises, the models predict both static properties (e.g., stiffness and deflections) and dynamic characteristics (natural frequencies, mode shapes), enabling systematic optimization and robust control design. The complexity of distributed compliance, geometric nonlinearities, and boundary conditions makes modeling challenging. Over the years, a range of theoretical methods has been proposed, from simplified analytical models to high-fidelity numerical simulations. This section emphasizes analytical and semi-analytical methods, focusing on their contributions to design trade-offs, system understanding, and performance evaluation.

### 3.1. Analytical and Semi-Analytical Modeling Techniques

**Figure 5:**
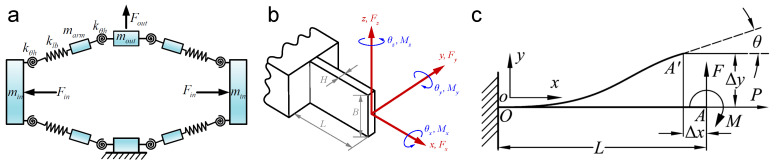
Analytical and semi-analytical modeling techniques. (**a**) The pseudo-rigid-body model [33]. (**b**) Stiffness matrix (compliance matrix) method. (**c**) Euler–Bernoulli beam theory [34].

Analytical and semi-analytical techniques build mathematical models from first principles of mechanics, yielding equations that describe a flexure’s static and dynamic behavior. These models provide deep physical insight and allow rapid parametric studies and optimization. However, to remain tractable, they usually rely on simplifying assumptions (e.g., small deflections, linear elasticity, rigid-body approximations), which may limit accuracy if a flexure operates in a highly nonlinear range. Nonetheless, analytical models have proven invaluable in the initial design and analysis phase of many nanopositioning stages, and advanced formulations continue to expand their validity range. Several widely used analytical modeling approaches are discussed below, as shown in Figure 5.

#### 3.1.1. The Pseudo-Rigid-Body Model

The pseudo-rigid-body model (PRBM) is a widely adopted semi-analytical method for modeling compliant mechanisms, including flexure-based nanopositioning stages. The central idea is to approximate distributed compliance by replacing flexible elements with an equivalent rigid-body mechanism consisting of rigid links and rotational springs [73,93,94,97,113]. Each flexure hinge or compliant beam is represented as a torsional spring of defined stiffness at a pivot, sometimes accompanied by an equivalent link length to emulate the deformed shape. This abstraction enables the direct application of classical rigid-body kinematics and dynamics to predict the mechanical response of compliant systems.

To ensure accuracy, PRBM parameters such as spring stiffness and link length are typically calibrated using experimental measurements or detailed nonlinear FEA. Once validated, the PRBM provides an efficient tool for design exploration. For instance, a PRBM-based dynamic model of a *Z*-axis compliant amplifier has been used to quickly assess displacement amplification and resonant frequency, showing strong agreement with FEA and experimental validation [93]. Similarly, the integration of PRBM with compliance matrix methods has been demonstrated for large-range remote center-of-motion flexures, yielding reliable predictions of stiffness characteristics and kinematic behavior.

The major advantage of PRBM lies in its ability to capture kinetostatic behavior with minimal computational effort. It can be effectively applied to estimate input forces for a desired displacement, evaluate amplification ratios, and quantify parasitic motions. Consequently, PRBM-based modeling has been successfully employed in numerous flexure mechanisms, such as XY microscanners, decoupling stages, and displacement amplifiers, providing valuable insights during preliminary design.

Nevertheless, the simplified nature of PRBM introduces certain limitations. Because mass and elasticity are lumped into discrete elements, higher-order vibration modes and distributed mass effects are not represented, which reduces accuracy for high-frequency dynamics or large deflections. As a result, while PRBMs can reliably predict quasi-static responses and fundamental frequencies, they may fail to capture detailed mode shapes or subtle stiffness variations at large displacements.

#### 3.1.2. Stiffness Matrix (Compliance Matrix) Method

The stiffness matrix method, closely related to the compliance matrix approach, is a widely used analytical tool in flexure mechanism design [28,54,97,114,115]. In this framework, each flexure element (e.g., beams, plates, hinges) is modeled with an elemental stiffness or compliance matrix [116,117]. These are then assembled into a global stiffness matrix K, which relates force and moment vectors to displacement and rotation vectors at structural nodes: F=Kx. By inverting K, or equivalently constructing a compliance matrix $C=K^{-1}$, the static deflections under given loads can be directly obtained. When combined with mass and inertia matrices, the method can also yield linearized dynamic responses.

A key advantage of this method is that it inherently accounts for the superposition of deformations in multiple flexure elements, while incorporating bending, axial, and shear compliance when supported by the element models. Under the assumption of linear elasticity and small deflections, the stiffness matrix approach offers high accuracy and computational efficiency, making it particularly suitable for computer-aided design and optimization. This efficiency enables designers to evaluate numerous geometric variations rapidly during early-stage exploration.

Applications of the method have demonstrated its effectiveness in predicting both static and dynamic behavior. For example, compliance matrix models have been employed to analyze large-range two-degree-of-freedom (DOF) rotation stages, capturing both kinematic characteristics and stiffness properties, with theoretical predictions validated against FEA and experimental prototypes [97]. Multi-DOF nanopositioning stages have also been modeled using 6×6 compliance matrices to evaluate stiffness and motion characteristics in each DOF, guiding the design of parallel flexure stages with centimeter-level travel. These studies show that the approach can accurately estimate load capacity, parasitic motions, and stiffness-displacement trade-offs in complex mechanisms.

Despite its strengths, the conventional stiffness matrix method is limited by its assumption of linearity. Large deflections introduce geometric nonlinearities that alter effective stiffness, requiring model extensions. Enhancements to this method include updating stiffness matrices as functions of displacement, deriving nonlinear stiffness matrices with geometric stiffness terms, or working directly in the compliance domain with higher-order terms and iterative solvers. Advanced formulations have been proposed to extend matrix methods to three-dimensional flexure geometries, integrating analytical compliance models of flexure hinges into a unified matrix framework. Such generalized models have been shown to achieve less than 20% error in predicting both static deflections and multiple modal frequencies of amplifier mechanisms, compared to high-fidelity 3D FEA.

The stiffness/compliance matrix method continues to serve as a backbone of flexure analysis, especially when combined with optimization algorithms such as genetic search or gradient-based techniques. Its efficiency facilitates automated multi-objective design, balancing criteria such as travel range, stiffness, resonance frequency, and parasitic error, which would be computationally prohibitive if using FEA alone. As a result, the method remains indispensable for both fundamental analysis and practical design optimization of compliant nanopositioning systems.

#### 3.1.3. Euler–Bernoulli Beam Theory for Flexure Hinges

Many flexure mechanisms can be effectively represented as assemblies of slender beams, making Euler–Bernoulli beam theory a fundamental analytical tool for deriving closed-form expressions of stiffness and deflection. The theory assumes that beam cross-sections remain plane and perpendicular to the neutral axis, thereby neglecting shear deformation and rotary inertia. These assumptions are valid for long and slender beams undergoing small angular deflections. Based on this formulation, one can derive the governing differential equation for beam deflection under load and solve for quantities such as tip deflection, slope, strain energy, and stress distribution. For common flexure geometries such as uniform beams, corner-filleted hinges, and right-circular hinges, the theory yields analytical formulas that directly relate stiffness to geometry. For example, the small-deflection rotational stiffness $K_{\theta }$ of a thin cantilevered beam with length *L*, width *b*, and thickness *t* can be estimated as $K_{\theta }\approx \frac{EI}{L}$, where *E* is Young’s modulus and $I=bt^{3}/12$ is the area moment of inertia.

The approach has been widely applied in flexure hinge design. Analytical models based on beam theory have been used to construct complete stiffness matrices for novel multi-DOF joints, enabling geometry optimization to maximize off-axis rigidity while satisfying resonance constraints [118]. Other studies have applied beam analysis to improve out-of-plane stiffness in XY parallel flexures, showing that appropriately arranged multi-beam configurations can achieve several times higher out-of-plane rigidity compared to conventional designs of similar range [42]. Such analytical estimates provide valuable design guidelines during the conceptual stage.

Beam theory can also be extended through energy methods such as Castigliano’s theorem to analyze more complex geometries. This approach has been used, for example, to derive closed-form load-deflection relationships for corner-filleted leaf-spring flexures under various boundary conditions [49]. The resulting formulas captured both bending stiffness and peak stress with high accuracy, and demonstrated how larger fillet radii can significantly reduce stress concentration with minimal stiffness loss. These insights provide direct guidance for enhancing fatigue performance in flexure-based systems.

Despite its usefulness, Euler–Bernoulli beam theory has notable limitations. By neglecting shear deformation and large rotation effects, it becomes inaccurate for short or thick flexures and for mechanisms operating at large deflections. In such cases, higher-fidelity models are necessary. Timoshenko beam theory, which incorporates shear flexibility, has been employed to model flexure strips with large width-to-length ratios, yielding compliance predictions within 5% of experimental data [112]. These results highlight that while simple beam models provide a solid foundation for estimating stiffness, resonance, and stress distribution, advanced formulations are required when shear and geometric nonlinearities dominate.

#### 3.1.4. Multibody Dynamics Approach

The multibody dynamics approach represents a flexure mechanism as an interconnected system of rigid bodies linked by flexible joints or elements [46,53]. Each body is assigned translational and rotational coordinates, while flexure hinges or beams are modeled as elastic constraints that store strain energy upon deformation. By applying analytical mechanics principles, such as Lagrange’s equations or Hamilton’s principle, the governing equations of motion can be derived, typically resulting in coupled nonlinear differential equations. This formulation naturally captures both large rigid-body motions and small elastic deformations, which makes it particularly well-suited for flexure mechanisms that simultaneously exhibit these behaviors.

Several studies have demonstrated the effectiveness of this approach. For instance, a five-degree-of-freedom hybrid flexure stage was modeled as a system of multiple rigid links connected by flexure hinge springs, with each hinge represented through a dynamic beam constraint model that contributed energy based on beam deformation [53]. The model successfully predicted the vibrational modes and revealed that a coiled flexure design could maintain near-constant stiffness over a large stroke, an observation later validated by experiments. Similarly, a compliant XYZ nanopositioning stage was developed using a multibody model that represented the stage as a rigid-body assembly constrained by flexures [46]. The framework guided the design of a millimeter-range stage capable of nanometric resolution, demonstrating the utility of multibody dynamics in practical nanopositioning stage development.

The strength of the multibody approach lies in its balance between accuracy and efficiency. Compared with lumped-mass models, it incorporates distributed compliance more realistically by modeling flexures as energy-storing constraints. At the same time, it is significantly more computationally efficient than full three-dimensional FEA, making it suitable for iterative design and optimization. This approach is especially powerful in analyzing parallel flexures or hybrid serial–parallel architectures, where motion in one substructure strongly influences others.

Recent advances have further expanded the capabilities of multibody modeling. For example, optimized compliant pivots with large range and negligible axis drift have been achieved by combining multibody kinetostatic formulations with optimization algorithms. These models have enabled systematic exploration of stiffness, cross-axis coupling, and frequency response, providing essential insights for control-oriented design.

#### 3.1.5. FEA-Based Methods

FEA is the most widely used high-fidelity modeling technique for flexure-based mechanisms [119,120,121,122,123,124]. In this method, the complex geometry of a flexure system is discretized into finite elements such as tetrahedra, bricks, shells, or beams, with each element governed by material constitutive relations. By assembling the global stiffness matrix, the continuum mechanics of the structure is approximated, and numerical solutions yield detailed predictions of displacement, stress, strain, and dynamic behavior. Owing to its generality, FEA can capture three-dimensional geometries, large deflections, contact conditions, and even material nonlinearities, making it a powerful tool for comprehensive analysis.

In practice, FEA is commonly employed to validate analytical models and verify final designs prior to fabrication. Numerous studies have used FEA modal and static analyses to confirm analytical predictions of resonance shifts, stiffness distribution, or load capacity in large-range flexure stages [5,97,98]. Across these examples, simulation results typically showed strong agreement with experimental data, with errors in stiffness and fundamental frequency often limited to a few percent. Modern commercial packages such as ANSYS, ABAQUS, and Nastran further enable coupled-field simulations, including piezoelectric actuation and thermal effects, which are critical for evaluating actuator-induced hysteresis or drift in nanopositioning systems.

The main drawbacks of FEA are its computational expense and reliance on specialized expertise. Constructing high-quality models requires careful attention to mesh quality, element selection, and boundary conditions, while optimization tasks remain challenging due to the high dimensionality and computational burden. Poorly defined meshes or constraints can easily yield inaccurate predictions, limiting its practicality for rapid design iteration. Despite these challenges, FEA remains the benchmark against which analytical models are compared. Many researchers adopt a hierarchical modeling strategy: analytical methods are used for preliminary design and parameter studies, while FEA is applied at later stages for high-fidelity validation. In addition, FEA results are often employed to calibrate simplified models, such as adjusting parameters in pseudo-rigid-body models or fitting coefficients in empirical stiffness formulas.

The choice of a modeling technique is a critical decision that impacts the design cycle, computational cost, and ultimate performance of the nanopositioning system. Each method offers a distinct set of trade-offs between accuracy, complexity, and computational expense, as summarized in Table 2.

### 3.2. Modeling Nonlinearities: Hysteresis and Creep

Flexure mechanisms are often well-approximated as linear elastic systems, provided material yielding is avoided and deflections remain moderate. However, when actuated by smart materials such as piezoelectric stacks, significant nonlinearities arise. Two critical effects are hysteresis and creep [64,80,100,101,109,125]. Hysteresis refers to the path-dependent extension of a piezoelectric actuator: under cyclic voltage excitation, the displacement follows a looped trajectory rather than a single-valued curve, which leads to positioning errors. Creep, on the other hand, is a time-dependent drift in position even under a constant input, typically manifesting over seconds to minutes due to viscoelastic relaxation. Although external to the flexure’s intrinsic mechanics, these nonlinearities critically affect nanopositioning accuracy and must be modeled to achieve high performance.

A variety of hysteresis models have been proposed. Physics-based approaches, such as Preisach and domain-wall models, attempt to represent the microscopic switching behavior of piezoelectric domains, while phenomenological approaches directly fit observed input-output relations. Among the latter, the Bouc–Wen model is widely used, producing loop-shaped hysteresis curves that can be tuned to match experimental data. Identified Bouc–Wen parameters are often exploited for feedforward linearization of actuator response. Other studies demonstrated that hysteresis and cross-coupling in multi-degree-of-freedom flexure stages introduce significant errors, which can be reduced below 1% through inverse rate-dependent compensators integrated into the control loop [126]. The Prandtl–Ishlinskii (PI) model is another popular option, representing hysteresis as a weighted sum of elementary operators with an analytically invertible form [127]. From a mechanics perspective, such models are typically integrated with the flexure’s linear dynamic model to yield hybrid actuator-structure representations [100,127].

Creep is commonly described by viscoelastic elements or empirical laws, such as logarithmic time dependence [128]. A Kelvin–Voigt model is often employed, where parameters are tuned to replicate observed long-term drifts. Although feedback control can mitigate creep in many cases, explicit creep models are essential for open-loop operation or accurate long-term prediction. Composite formulations have also been proposed, combining a Bouc–Wen hysteresis kernel with exponential decay terms for creep, thereby capturing both instantaneous and time-dependent nonlinearities [101]. Modern controllers sometimes embed dedicated creep compensators based on such formulations.

In summary, while the flexure mechanism itself remains linear, actuator-induced hysteresis and creep necessitate dedicated modeling. Depending on the application, hysteresis and creep models may be incorporated into the overall nanopositioning stage dynamics to predict and compensate for errors. The trade-off between accuracy and invertibility often guides the model choice: the Bouc–Wen model offers strong flexibility but limited analytical invertibility, whereas the PI model provides straightforward inversion at the expense of generality. Importantly, these models are identified from experimental data and represent actuator behavior rather than structural elasticity. This underscores that complete nanopositioning stage models must integrate elastic mechanics with actuator (and sometimes sensor) dynamics. Recent advances also explore data-driven approaches, such as machine learning, to capture hysteresis without assuming a specific model structure. While promising, such methods remain beyond the present scope; for most applications, well-established hysteresis and creep models are sufficient to achieve nanometer-level accuracy.

### 3.3. Model Validation and System Identification

Developing a theoretical model is only the first step; it is equally critical to validate the model against experimental results. Flexure-based systems are often custom-designed, and small errors can arise from unmodeled parasitic flexibilities, manufacturing deviations, or uncertainties in material properties. Therefore, model validation is routinely performed by comparing analytical or simulated predictions with measurements obtained from physical prototypes. Key validation metrics include static stiffness, where deflection under known loads is compared to model predictions, and dynamic response, where resonant frequencies and mode shapes are measured through modal testing.

Several studies have demonstrated this process of validation. For example, one prototype of a long-travel XY stage was built and tested, with resonance frequencies and cross-coupling measured experimentally. The first mode was found to be close to 86 Hz, confirming the accuracy of the theoretical prediction [5]. Similarly, in another study, a flexure stage’s step response and frequency response were measured, showing excellent agreement with model predictions, with steady-state gain and natural frequency matching within a few percent [93]. When discrepancies arise, they typically indicate the need for refinements, such as including neglected compliances or adding damping estimates if dynamic amplitudes are over-predicted.

Experimental modal analysis (EMA) is a widely adopted approach for dynamic validation. In EMA, the structure is excited by an instrumented hammer or shaker, and the response is measured using accelerometers or laser vibrometers. The resulting frequency response functions are curve-fit to extract modal frequencies, damping ratios, and mode shapes, which can be directly compared with eigen-analysis predictions. Many flexure mechanism studies summarize these results in tabulated comparisons of predicted versus measured modal frequencies to illustrate model accuracy.

In cases where analytical predictions deviate significantly, system identification techniques are employed. This approach treats the nanopositioning stage as a “black-box” or “gray-box” system, fitting a dynamic model to measured input-output data. For instance, a piezo-driven stage may be excited with a broadband signal, and the resulting response is used to fit a high-order transfer function model that captures additional unmodeled dynamics. By comparing the identified model with the physics-based formulation, designers can detect unmodeled high-frequency modes or actuator effects.

Practical design cycles often follow an iterative process: analytic models guide the design, prototypes are built, experimental testing reveals discrepancies, and the models are refined accordingly. These refined models then serve as a foundation for controller design. For example, one study developed a flexure stage model, validated it experimentally to identify uncertainties and coupling, and subsequently designed a disturbance-observer-based controller that explicitly accounted for those uncertainties, achieving sub-nanometer tracking precision [126]. In another case, a cross-coupling compensation algorithm for an AFM scanner was implemented based on an accurate model of the coupling dynamics between fast and slow axes; compensation performance improved markedly once the model was experimentally verified and tuned [9].

Rigorous validation of theoretical models against experimental data is indispensable for ensuring trustworthy analysis and design. Techniques such as static load testing, frequency response measurement, and EMA should complement any modeling effort. When mismatches occur, system identification helps refine model parameters or uncover missing dynamics. Once validated, the refined models provide a reliable foundation for design optimization and control development. The literature consistently emphasizes this cycle of modeling, prediction, experimental validation, and refinement, which has become a cornerstone in the development of high-performance flexure-based nanopositioning stages. By ensuring that theoretical models closely reflect reality, designers can confidently pursue larger ranges, higher speeds, and nanometer-level precision in integrated nanopositioning stages. A high-fidelity model is not an end in itself but rather the essential foundation upon which advanced control systems are built. The accuracy of these models directly dictates the performance and robustness of the control strategies employed to counteract inherent system dynamics and disturbances.

## 4. Control Strategies

**Figure 6:**
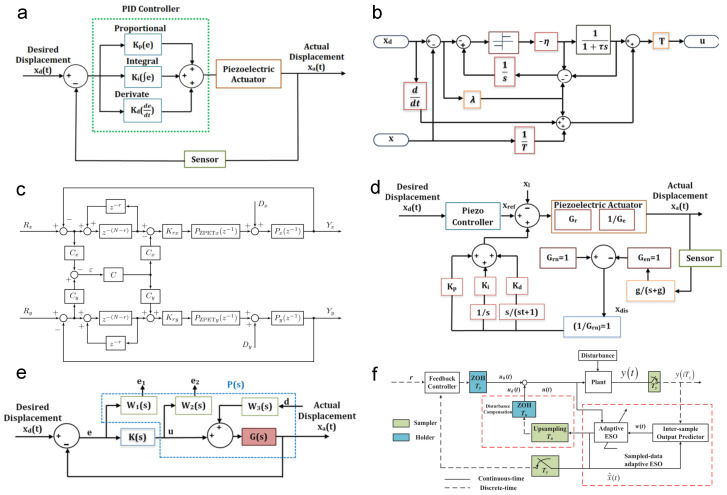
Control strategies. (**a**) PID controller [35]. (**b**) Sliding mode control [35]. (**c**) Repetitive controller with a cross-coupled controller [36]. (**d**) Disturbance observer-based control [35]. (**e**) $H_{\infty }$ control [35]. (**f**) Extended state observer-based control [37].

Modern flexure-based nanopositioning stages demand control systems that can achieve nanometer precision, high bandwidth, and robust stability despite challenges such as lightly damped resonances, actuator nonlinearities, and multi-axis coupling. A variety of control strategies have been developed to meet these demands, ranging from classical feedback loops to advanced hybrid algorithms. This section reviews these strategies in a structured manner, highlighting how each addresses key issues like precision trajectory tracking, cross-axis decoupling, bandwidth enhancement, and disturbance rejection. The discussion is organized into subsections on active resonant control, classical control, robust control, adaptive control, learning-based methods, and multi-axis contour tracking control, as illustrated in Figure 6. Each subsection synthesizes findings from the literature, with an emphasis on representative examples and performance achievements.

### 4.1. Active Resonant Control

The lightly damped resonant characteristics of flexure-based mechanisms significantly limit their motion speed and precision. Active resonant control methods address this fundamental challenge by introducing positive feedback to modify the system’s pole-zero distribution, thereby increasing the damping ratio and suppressing mechanical resonances. Various damping control techniques have been developed, including resonant controller [129], integral resonant control (IRC) [130,131,132], integral force feedback (IFF) [133], positive position feedback (PPF) [134], positive velocity and position feedback (PVPF) [135,136], and positive acceleration, velocity and position feedback (PAVPF) [137,138].

**Figure 7:**
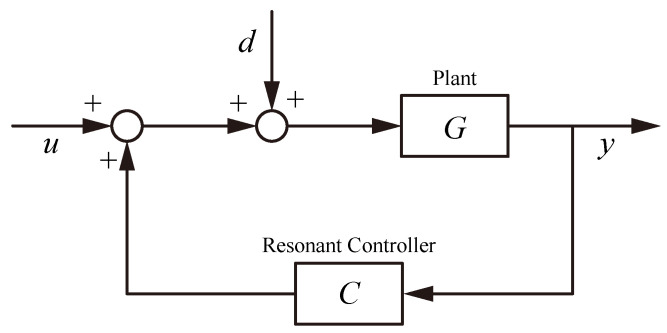
Block diagram of the active resonant control.

A general block diagram illustrating the typical configuration of active resonant control is shown in Figure 7, which provides a conceptual foundation for the specific control strategies discussed below. Early developments focused on fundamental positive feedback strategies. Moheimani et al.completed the experimental implementation of an extended multivariable PPF controller, establishing its practical viability for active structures [134]. Building upon this, Fleming et al. conducted work on the simultaneous optimization of damping and tracking controller parameters via selective pole placement for PVPF control, demonstrating enhanced positioning bandwidth for nanopositioning stages [135]. Further advancing this lineage, Zhu et al. accomplished the development of a PAVPF-based damping control approach, analytically enabling arbitrary pole placement for a third-order model [138]. Aphale et al. subsequently performed experimental validation of a simultaneous design strategy for PAVPF control, achieving a three-fold increase in closed-loop bandwidth compared to traditional sequential design methods [137].

Alternative resonant control strategies have also been extensively explored. Moheimani et al. implemented an IRC scheme using a field-programmable analog array (FPAA) for a high-speed AFM nanopositioning stage, demonstrating effective multi-mode damping [131]. In the domain of advanced control, Aphale et al. designed a novel two-degrees-of-freedom PI^2^D controller that replaces the integral action with a double integral, successfully overcoming stability limitations associated with hardware-induced time delays for precise nanopositioning [139]. Further advancing the design methodologies for positive feedback controllers, Aphale et al. completed a linear matrix inequality (LMI)-based design framework for PPF, PVPF, and PAVPF controllers, providing a convex optimization approach to eigenstructure assignment for enhanced damping performance [140].

In summary, these diverse active resonant control methods effectively enhance the system damping ratio, thereby alleviating low gain margin issues and providing a critical foundation for resonance suppression necessary for high-precision trajectory tracking. Nevertheless, the design and implementation of controllers such as PPF, PVPF, and PAVPF entail specific considerations. The design process generally requires system identification to obtain a transfer function model of the lightly damped plant. A dedicated damping controller must then be developed for each vibrational mode. In multi-mode systems, this results in a controller complexity that scales with the number of modes. Since accurately identifying higher-order modes is often challenging and their amplitudes are generally significantly smaller than that of the fundamental mode, it is common practice to approximate the system as a second-order model, with damping control applied primarily to the dominant mode. Although Li et al. [138] demonstrated successful application of a PAVPF controller to a third-order system, extending this family of damping strategies to general higher-order or non-minimum phase systems remains an open challenge. Despite these practical constraints, the progression from basic PPF to more advanced architectures reflects a continued effort toward achieving higher bandwidth and improved robustness in nanopositioning systems.

### 4.2. Tracking Control

Tracking control plays a pivotal role in nanopositioning systems, directly determining their ability to follow desired trajectories with nanometer or even sub-nanometer precision. Effective tracking is essential for applications such as high-resolution lithography, surface metrology, and nanomanipulation, where both dynamic accuracy and stability are critical. The design of a tracking controller aims to minimize position error, maintain robustness against system uncertainties, and ensure smooth motion across a wide operating bandwidth. This subsection reviews key techniques commonly employed in nanopositioning, including PID regulation, loop-shaping compensators, and feedforward strategies, highlighting their principles and effectiveness in achieving high-precision motion control.

**Figure 8:**
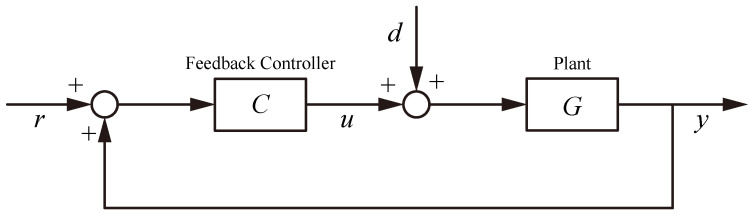
Block diagram of the feedback control.

PID regulation serves as the foundational feedback mechanism in most nanopositioning stages owing to its simplicity and reliability. The basic feedback architecture is illustrated in Figure 8, providing a reference for subsequent enhancements. Proportional-Integral-Derivative (PID) regulators are particularly ubiquitous in industrial nanopositioning systems [78,80,141,142]. Properly tuned PID controllers provide stable regulation and sub-nanometer steady-state accuracy by continuously correcting position error. Shiou et al. [125] implemented a real-time PID-based closed-loop system on an FPGA-driven piezo stage, achieving ±2 nm steady-state precision over a 120 µm range. Similarly, Li et al. [80] demonstrated that an XY flexure stage equipped with PID feedback achieved nanometric contouring performance, significantly outperforming open-loop operation which suffered from hysteresis and drift. These implementations underscore how even straightforward PID control can dramatically improve precision when sufficient loop gain is maintained across the operating bandwidth.

To further enhance the performance of basic PID regulation, loop-shaping techniques are frequently adopted. Lead-lag compensators and notch filters represent two widely used approaches for modifying the system’s frequency response. Lead compensation adds phase boost near lightly damped resonant frequencies, thereby increasing stability margins. Li et al. [143] applied lead compensation to a reluctance-actuated nano-manipulator, effectively stabilizing the system prior to implementing finer control actions. Notch filters are specifically employed to suppress resonant peaks, enabling higher usable bandwidth without sacrificing low-frequency gain. Cai et al. [101] designed sophisticated compensators for a six-degree-of-freedom piezo stage, achieving a first resonant frequency of 586 Hz while maintaining wide stability margins and cross-coupling suppression below 1.7%.

Feedforward compensation is often integrated with feedback control to further enhance tracking precision and dynamic response. The combination of PID feedback for stability and disturbance rejection with feedforward components for reference shaping forms an effective composite control architecture. Chen et al. [144] developed a dual-drive XY stage that combined a coarse voice-coil actuator with a fine piezo actuator, allocating large-range motion to the coarse drive and high-frequency corrections to the fine drive through an optimized feedforward schedule. This dual-servo scheme achieved a 3 mm × 3 mm range with micrometer-level accuracy. Other systems incorporate direct inverse models in the feedforward path to linearize actuator-stage behavior, yielding substantial accuracy improvements without requiring complex modeling approaches.

In summary, tracking control methods—including PID regulators, loop-shaping compensators, and feedforward strategies—form the backbone of many practical nanopositioning systems. Their principal advantages lie in straightforward implementation, reliable performance, and well-established design methodologies, enabling high steady-state accuracy and reasonable bandwidth for systems with well-behaved dynamics. However, these approaches face limitations when dealing with significant nonlinear effects such as hysteresis and creep, high-frequency resonant modes, and substantial model uncertainties. As the demands for faster scanning, larger travel ranges, and sustained sub-nanometer accuracy increase, these tracking control frameworks often require integration with more advanced control techniques to achieve comprehensive tracking performance.

### 4.3. Robust Control

Robust control strategies play a pivotal role in nanopositioning systems by ensuring stability and performance in the presence of model uncertainties, external disturbances, and parameter variations. These methods are particularly valuable for flexure-based nanopositioning stages, which often exhibit variations in material properties, changing load conditions, and complex unmodeled dynamics. This subsection examines several prominent robust control approaches, including disturbance observer-based (DOB) methods [145], sliding mode control (SMC) [146], frequency-domain techniques [147,148], active disturbance rejection control (ADRC) [149], highlighting their respective applications and effectiveness in addressing nanopositioning challenges.

DOB-based methods provide an effective framework for real-time estimation and compensation of external perturbations and unmodeled dynamics without requiring exact disturbance models. Feng et al. implemented a DOB-based control scheme that estimated force disturbances on the fine stage caused by coarse stage motion in a dual-stage nanopositioning system, achieving nanoscale accuracy across a 5 mm range [145]. These methods effectively handle system uncertainties by treating them as equivalent disturbances, enabling robust performance under varying operating conditions.

SMC offers remarkable insensitivity to parameter variations and bounded nonlinearities, making it particularly suitable for nanopositioning applications with uncertain dynamics. Aphale et al. developed a PID-type sliding surface to regulate a 2-DOF flexure stage under varying payloads, maintaining resolution below 250 nm over a 2 mm stroke [146]. Although classical SMC suffers from chattering phenomena, practical implementations employ smoothing functions or high-bandwidth actuators to mitigate this limitation while preserving robustness.

Frequency-domain robust methods, particularly $H_{\infty }$ control and μ-synthesis, provide systematic frameworks for addressing structured and unstructured uncertainties. Aphale et al. designed a robust anti-windup controller through $H_{\infty }$ optimization that ensured stability of a piezo stage even during actuator saturation [147]. Extending beyond conventional $H_{\infty }$ methods, Ahmad et al. completed the development of a μ-synthesis robust feedback controller combined with a Dahl model-based feedforward compensator, effectively addressing both hysteresis nonlinearity and system model uncertainties [148]. This integrated approach demonstrated significant improvements, achieving 95% hysteresis compensation and 80% tracking error reduction, showcasing μ-synthesis’s capability in handling complex uncertainty structures.

Active disturbance rejection control (ADRC) employs extended state observers to lump unmodeled dynamics and external perturbations into unified disturbance estimates. Yang et al. applied ADRC to a stiffness-adjustable stage, treating varying stiffness and nonlinearities as lumped disturbances [149]. This approach achieved faster settling times and improved stability across different stiffness levels compared to conventional PID control, demonstrating ADRC’s effectiveness in handling complex, time-varying uncertainties.

In summary, robust control strategies provide essential capabilities for maintaining nanopositioning performance under realistic operating conditions characterized by uncertainties and disturbances. Their principal advantages include formal guarantees of stability robustness, systematic handling of various uncertainty types, and the ability to maintain performance across a prescribed range of system dynamics. However, these methods often involve increased computational complexity, require more sophisticated design procedures, and may lead to conservative performance when uncertainty bounds are overly pessimistic. As nanopositioning applications continue to demand higher precision under increasingly challenging conditions, a promising path forward is the coordinated integration of robustness-enhancing and high-precision techniques, ensuring that nanometer-scale accuracy is both achieved and maintained.

### 4.4. Adaptive and Learning-Based Control

Adaptive and learning-based control strategies represent a paradigm shift in nanopositioning, enabling systems to autonomously adjust to uncertainties, time-varying dynamics, and complex nonlinearities that challenge fixed-parameter controllers [44,109,143,150,151,152,153,154]. These approaches can be broadly categorized into model-based adaptive techniques, which explicitly incorporate system models into the adaptation logic, and data-driven learning methods, which improve performance directly from operational data. This subsection examines key developments in both categories, highlighting their applications in addressing critical challenges such as parameter variations, actuator saturation, repetitive tracking, and unmodeled dynamics in precision motion systems.

Model-based adaptive methods leverage varying degrees of system knowledge to achieve robust performance under uncertainties. Model reference adaptive control (MRAC) stands as one of the most established approaches, particularly effective when system parameters are partially known. Ling et al. developed a model reference adaptive integral resonant control (MRAIRC) scheme for a piezo-actuated nanopositioning stage, where adaptive laws continuously adjusted controller gains to compensate for load variations from 0 to 1000 g [150]. This approach maintained superior tracking performance across different scanning frequencies compared to standard IRC with fixed gains. Extending this concept, Xu et al. created a model reference adaptive control with perturbation estimation (MRACPE) scheme that treated unmodeled dynamics and nonlinearities as lumped perturbations, achieving predesigned tracking error bounds and significantly enlarged control bandwidth for both set-point and sinusoidal positioning tasks [151].

Adaptive control also proves valuable for managing actuator saturation and large-range operations. Liu et al. combined an internal-model-based tracking controller with an $H_{\infty }$-optimized anti-windup compensator featuring adaptive elements to guarantee stability during saturation conditions in an XYZ piezo stage [44]. This hybrid approach maintained trajectory errors below 0.3% even at travel limits, with the adaptive component automatically tuning anti-windup gains based on saturation levels. Similarly, gain scheduling strategies have been effectively deployed for systems with variable dynamics, such as stiffness-adjustable flexure stages where ADRC parameters are adapted in real-time based on stiffness settings to maintain consistent performance across operating extremes [152].

Learning-based methods excel in applications involving repetitive operations or where accurate system models are difficult to obtain. Iterative learning control (ILC) and repetitive control (RC) have demonstrated remarkable success in periodic motion tasks by leveraging historical error data to refine subsequent control actions. Li et al. implemented a model-free adaptive ILC (MFAILC) on a large-range reluctance-actuated nanopositioning stage, reducing triangular-wave tracking error from several hundred nanometers to just 66 nm through successive learning cycles. Advanced variants include modified repetitive control (MRC) with embedded infinite impulse response filters to enhance robustness against non-periodic disturbances while maintaining precise periodic tracking capability [9].

Machine learning techniques have further expanded the capabilities of data-driven control. Wu et al. employed a multilayer feedforward neural network as a hysteresis compensator for an XYθ stage, where the network learned the inverse hysteresis mapping directly from experimental data [109]. Yin et al. recently demonstrated an LSTM-based inverse model combined with model predictive control that achieved broadband precision tracking across multiple axes [155]. When integrated with PID feedback, this approach achieved spiral and epicycloid trajectory tracking with root-mean-square errors of only tens of nanometers. Reinforcement learning (RL) has also been explored for nanopositioning, though current implementations remain primarily in simulation due to computational demands and safety considerations during the exploration phase [153].

In summary, adaptive and learning-based control methods provide powerful capabilities for maintaining high precision under uncertain and time-varying conditions. Their principal advantages include the ability to automatically compensate for parameter variations, adapt to changing operating regimes, and progressively improve performance through iteration or experience. However, these benefits come with increased design complexity, potential stability challenges if adaptation mechanisms are overly aggressive, and substantial computational requirements—particularly for data-intensive learning approaches. As computational resources advance and learning algorithms become more efficient, the integration of adaptive and learning elements is expected to play an increasingly vital role in achieving the next generation of high-precision, self-optimizing nanopositioning systems.

### 4.5. Multi-Axis Contour Tracking Control

Multi-axis contour tracking control addresses the critical challenge of achieving precise coordinated motion across multiple axes in nanopositioning systems, where the relative accuracy between axes often determines the overall contouring performance [36,156,157,158,159,160,161,162]. Unlike single-axis control that focuses solely on individual axis tracking error, contour control explicitly minimizes the deviation from the desired path, making it essential for applications such as micro/nano-fabrication, scanning probe microscopy, and high-precision machining. This subsection reviews major contour control methodologies, including cross-coupled control strategies, task coordinate frame approaches, and position-domain control techniques, highlighting their respective mechanisms for enhancing multi-axis coordination precision.

Cross-coupled control (CCC) represents one of the most fundamental approaches to contour tracking, introducing coordination between axes by calculating contour error from individual axis errors. Koren’s pioneering work established the basic CCC framework with constant gains, though its applicability was limited to simple contours [156]. Subsequent developments led to variable-gain CCC that approximates free-form contours as circular arcs, with gains adjusting according to trigonometric ratios for improved performance. Further extending this concept, Huo et al. developed generalized CCC (GCCC) for irregular shapes by computing contour error as the distance from the actual position to the line connecting the two nearest reference points [157]. Advanced hybrid approaches combine CCC with learning techniques; Ulu et al. created a modular learning-based cross-coupled controller that integrates contour error estimation with iterative learning for arbitrary nonlinear contours, achieving nanometer-level precision [158]. Similarly, Barton et al. formulated cross-coupled ILC (CCILC) by reformatting multi-axis CCC into a single-input single-output ILC framework, enabling systematic learning of contour errors alongside individual axis tracking [159].

Task coordinate frame (TCF) methods address contour control through coordinate transformation that decouples error dynamics into tangential and normal components. Tomizuka et al.established the foundational TCF approach by transforming machine tool feed drive dynamics into a moving coordinate frame attached to the desired contour [160]. This transformation revealed the effects of contour curvature and feed rate on control performance, leading to a control law comprising linear time-varying PD feedback and trajectory feedforward components. Experimental validation demonstrated significant contouring accuracy improvement and successful decoupling between tangential and normal dynamics. The generalized TCF (GTCF) framework later provided precise first-order approximation of contour error regardless of absolute error magnitude, with adaptive robust control in individual axes further enhancing precision and robustness [161].

Position domain control (PDC) represents a paradigm shift by treating one axis displacement as the independent variable rather than time. This event-driven approach transforms contour tracking into position-domain function tracking, offering superior performance compared to traditional time-domain methods. Recent innovations have combined PDC with learning-based cross-coupled control, integrating the advantages of both approaches. Further advancing time-domain approaches, Zhang et al. developed a robust time-varying internal model principle-based control (TV-IMPC) with gray-box extended state observer for sophisticated reference tracking, achieving approximately 60 nm RMSE over a ±80 mm stroke in a direct-drive servo stage [162]. For repetitive applications, the same authors implemented a cross-coupled repetitive control structure that integrates RC for periodic trajectory tracking with CCC for contour error coordination, achieving 86 nm contour error in micro-stereolithography applications [36].

In summary, multi-axis contour tracking control strategies provide essential capabilities for applications requiring precise coordinated motion across multiple axes. The principal advantages of these approaches include explicit minimization of contour error rather than individual axis errors, adaptability to complex contour geometries, and compatibility with various advanced control techniques such as iterative learning and adaptive robust control. However, these methods typically involve increased computational complexity, require accurate system models for effective implementation, and may introduce stability challenges when multiple control loops interact. As nanopositioning applications continue to demand higher precision in complex multi-axis trajectories, the integration of different contour control paradigms—particularly combining learning-based approaches with robust control frameworks—represents a promising direction for achieving both high precision and robustness in next-generation multi-axis systems.

### 4.6. Summary

The preceding subsections have provided a detailed examination of the principal control strategies employed in flexure-based nanopositioning systems. As discussed, these methods—ranging from active resonant damping and classical feedback to robust, adaptive, and multi-axis contour tracking approaches—each address specific challenges such as vibrational resonances, model uncertainties, nonlinearities, and the critical need for coordinated motion. To synthesize this extensive review and provide a clear, comparative overview of their core attributes, the fundamental characteristics of these strategies are systematically contrasted in Table 3. This comparison highlights their distinct operational mechanisms, primary advantages, and inherent limitations.

In conclusion, this section has delineated the landscape of control methodologies available for high-precision nanopositioning. The selection of an appropriate strategy is fundamentally contingent upon the specific performance requirements—including the required bandwidth, precision, and operational range—coupled with the inherent dynamics of the system to be controlled and the practical constraints of the implementation stage. No single approach constitutes a universal solution; rather, the optimal choice involves a critical trade-off between the simplicity and reliability of classical methods and the enhanced performance and robustness offered by more advanced, albeit complex, strategies. Despite the sophistication of existing design, modeling, and control techniques, the relentless pursuit of greater performance exposes fundamental limitations and new challenges. Addressing these emerging frontiers requires a critical look at persistent trade-offs and the integration of transformative new approaches.

## 5. Challenges and Future Outlook

Despite significant advancements, flexure-based nanopositioning technology continues to face several fundamental challenges that limit its performance in emerging applications demanding higher speeds, larger ranges, and greater precision. This section critically examines these persistent hurdles, with a particular focus on the paramount challenge of dynamic control, and outlines promising research directions aimed at overcoming these limitations. A critical analysis of trade-offs and the integration of modern, data-driven techniques are emphasized as key to future progress.

### 5.1. High-Bandwidth Control for Flexure-Based Nanopositioning Stages

The pursuit of higher operating bandwidths is a primary driver in nanopositioning research, directly impacting throughput in applications like high-speed AFM and semiconductor inspection. However, this pursuit is fundamentally constrained by the flexure stage’s lightly damped resonant dynamics, which create a severe trade-off between speed and stability. As target bandwidths push into hundreds of hertz, the low-frequency structural modes become the dominant performance bottleneck, limiting achievable control bandwidth and inducing tracking errors. This challenge is exacerbated in multi-DOF stages where complex mode shapes lead to significant cross-axis coupling. While passive damping is often ineffective at the nanometer scale, active resonant control strategies have emerged as the indispensable solution, employing sophisticated feedback to inject damping and reshape the system’s frequency response. Nevertheless, these very strategies face significant limitations that must be overcome to unlock the next generation of high-bandwidth nanopositioning stages.

A family of positive feedback techniques forms the cornerstone of active damping. IRC offers a simple, robust means of damping the dominant resonant mode by feeding back a filtered version of the position signal through an integral term, effectively creating a virtual damper [130,131]. PPF uses a second-order compensator tuned to the target resonance, providing phase lead that increases the damping ratio [134]. Building on this, PVPF and its extension, PAVPF, incorporate additional state feedback to enable arbitrary pole placement for higher-order models, offering greater design flexibility for multi-mode damping [135,137,138]. The implementation of these controllers, particularly for multi-mode systems, has been advanced significantly by convex optimization frameworks such as LMIs, which facilitate robust and simultaneous design of damping and tracking controllers [140].

Despite these advances, critical limitations hinder their widespread effectiveness in high-bandwidth applications. The performance of these methods is highly sensitive to the accuracy of the identified system model. Inaccurate identification of resonant frequencies and mode shapes can lead to reduced performance or even instability. Furthermore, applying these techniques to systems with numerous, closely-spaced modes is non-trivial, often resulting in complex, high-order controllers that are difficult to implement robustly. There is a critical trade-off between the complexity of the controller (and thus its robustness to model uncertainty) and the degree of performance enhancement achieved. Future research must focus on developing adaptive resonant control strategies that can autonomously track changes in system dynamics due to factors like payload variation or thermal drift, ensuring consistent high-performance operation.

### 5.2. Critical Trade-Offs and Performance Boundaries

The design of nanopositioning stages is inherently an exercise in managing trade-offs. A central, unresolved challenge is the conflict between range, precision, and bandwidth. Maximizing travel range typically requires more compliant flexures, which reduces the structural stiffness and hence the resonant frequency, thereby limiting the control bandwidth. Conversely, designing for high bandwidth (high stiffness) constrains the maximum elastic deformation and thus the range. Innovations like displacement amplification and hybrid coarse-fine actuation attempt to circumvent this trade-off, but they introduce their own complexities, such as added mass, reduced rigidity, and control coupling between stages. There is a fundamental physical limit to the product of range and bandwidth for a given actuator technology and material, and current designs are steadily approaching this boundary. Future breakthroughs may rely on fundamentally new materials with higher strength-to-stiffness ratios or metamaterials with tailorable compliance.

Another critical trade-off exists between modeling fidelity and computational tractability. High-fidelity physics-based models (e.g., nonlinear FEA) provide excellent accuracy but are too computationally intensive for real-time control or rapid design optimization. Conversely, simplified analytical models (e.g., PRBM, beam theory) are computationally efficient but may lack the accuracy required for predicting complex behaviors like parasitic motions or nonlinear stiffness. This gap is particularly evident in the control of multi-axis systems, where simplified decoupled models fail to capture dynamic cross-coupling effects, leading to degraded contouring accuracy. The challenge is to develop reduced-order models that retain critical physical insights and dynamics while being sufficiently lightweight for control and optimization. Here, data-driven modeling techniques such as system identification and machine learning offer a promising path forward by creating accurate dynamic models directly from experimental data, potentially bridging the fidelity-efficiency gap.

### 5.3. Embracing Data-Driven and AI-Enhanced Methodologies

A significant paradigm shift in nanopositioning research involves the growing adoption of data-driven and artificial intelligence (AI) methodologies to overcome the limitations of traditional model-based approaches. While physics-based models provide a foundational understanding, their effectiveness is often constrained by difficulties in accurately capturing complex, rate-dependent nonlinearities such as hysteresis and creep, as well as unmodeled dynamics and time-varying disturbances. This limitation has spurred the exploration of techniques that leverage operational data to learn system behavior directly, paving the way for model-free or hybrid solutions that enhance performance, adaptability, and robustness. The application of machine learning for modeling intricate dynamics is a key area of progress. For instance, Gaussian process (GP) regression has been effectively used to model rate-dependent hysteresis, with advanced variants like the frequency-separation-based Gaussian process (FSGP) improving computational efficiency by selectively using training data relevant to the target operational frequency, enabling effective compensation in high-speed applications such as atomic force microscopy [163]. Beyond pure modeling, a promising direction is the integration of data-driven techniques with established model-based frameworks. This hybrid approach is exemplified by enhancing the zero-phase error tracking feedforward control (ZPETFC) strategy through data-based optimization, where methods like instrumental-variable estimation are used to fine-tune compensators, achieving robust nanometer-level tracking accuracy even for complex, non-minimum phase systems [164].

Further advancing control capabilities, AI techniques are being deeply integrated into the control loop itself. Neural networks, particularly gated recurrent units (GRUs), have demonstrated a remarkable ability to predict tracking error dynamics from historical data. These predictions can be incorporated into frameworks like learning adaptive robust control (LARC) to generate proactive feedforward compensation, significantly improving transient and steady-state performance while maintaining robustness against uncertainties [165]. Similarly, intelligent feedforward prediction schemes construct compensation signals based on the learned error characteristics of a system under high-bandwidth control, enabling precise tracking of high-frequency trajectories [166]. The scope of these intelligent methods also extends to challenging operational scenarios, such as networked control systems where issues like signal quantization and actuator failures are present. In these contexts, event-triggered neural control strategies incorporating specialized filters have been developed to maintain stability and performance despite rate-dependent hysteresis nonlinearities [167].

Despite the promising advances, the wider adoption of data-driven and AI-enhanced methodologies faces significant challenges. The “black-box” nature of many algorithms raises concerns regarding predictability and stability guarantees in safety-critical applications. Furthermore, the computational complexity of sophisticated models can be prohibitive for real-time implementation on embedded stages. The future outlook points toward the development of hybrid intelligent control frameworks that synergistically combine the interpretability and stability guarantees of model-based control with the adaptability and high performance of data-driven techniques. Critical research thrusts will need to focus on creating explainable AI methods for control systems, designing real-time learning algorithms with formal stability guarantees, developing efficient software frameworks for seamless integration, and exploring hardware acceleration solutions to meet the stringent latency requirements of high-bandwidth nanopositioning systems.

### 5.4. Integration and Co-Design as a Path Forward

The conventional sequential design process—where the mechanical structure is optimized first, followed by the selection of actuators and sensors, and finally the design of the control system—is increasingly recognized as a fundamental barrier to achieving optimal performance in nanopositioning systems. This approach inherently limits the exploration of synergistic solutions where the mechanical, electrical, and control domains can mutually enhance each other. The future of high-performance nanopositioning, therefore, lies in the adoption of a holistic mechatronic co-design philosophy. This paradigm treats the mechanical structure, actuator dynamics, sensor placement, and control algorithm as a single, coupled optimization problem. For instance, the flexure topology can be optimized not only for high stiffness and kinematic decoupling but also to shape the system’s frequency response, making it inherently more amenable to high-bandwidth control by passively separating resonant modes or reducing cross-coupling. Similarly, the integration of sensing directly into the flexure structure—such as using embedded strain gauges or leveraging the self-sensing capabilities of piezoelectric actuators—can minimize measurement delays and reduce noise, effectively increasing the achievable control bandwidth. Additive manufacturing is a key enabler of this co-design approach, allowing for the fabrication of complex, topology-optimized, and multi-functional structures that are impossible to produce with traditional subtractive machining, thus unlocking previously inaccessible regions of the design space.

Expanding beyond the traditional scope of mechatronics, co-design must further embrace multifunctional integration and multi-physics optimization. This involves the exploration of smart material systems, such as designing flexures with spatially tailored compliance using functional gradients or integrating piezoelectric materials directly into the structure for simultaneous actuation and sensing. Furthermore, the thermal management of actuators, which is a critical source of drift and instability in precision systems, must be considered from the outset. A co-design approach could, for example, optimize the structural geometry to enhance heat dissipation or incorporate integrated cooling channels. The ultimate goal is to evolve from designing a collection of components to engineering a unified cyber-physical system where the physical mechanism and the digital controller are deeply intertwined. Advanced model-based design frameworks, incorporating high-fidelity simulations of multi-physics interactions (structural, electrical, thermal), are crucial for exploring these complex trade-offs. By simultaneously optimizing across disciplinary boundaries, co-design promises to deliver nanopositioning systems that are not only faster and more precise but also more compact, energy-efficient, and robust to operational variations, thereby meeting the escalating demands of next-generation applications in fields like quantum technology and advanced semiconductor metrology.

### 5.5. Future Horizons

The field of flexure-based nanopositioning is at a pivotal juncture. While existing technologies have matured to enable remarkable precision, the demands of next-generation applications in quantum technology, bio-nanotechnology, and advanced semiconductor manufacturing will require a new level of performance. Addressing the core challenge of dynamic control through advanced resonant damping strategies, honestly confronting the fundamental performance trade-offs, and fully embracing the potential of data-driven co-design are essential steps forward. The integration of smart materials, additive manufacturing, and AI-driven control promises to usher in a new era of nanopositioning systems that are not only faster and more precise but also more adaptive, robust, and compact. The future outlook is bright, contingent upon a multidisciplinary research approach that breaks down the traditional barriers between mechanics, electronics, and computer science.

## 6. Conclusions

This review has provided a comprehensive overview of flexure-based nanopositioning stages, emphasizing the interconnected roles of design, modeling, and control strategies in achieving high-precision motion. From foundational design principles and structural topologies to advanced optimization and co-design methodologies, the discussion has highlighted both the strengths and limitations inherent in current technologies.

Flexure-based mechanisms continue to stand out for their frictionless, backlash-free operation and exceptional resolution. However, overcoming challenges such as parasitic motion, limited travel range, and nonlinear dynamic behavior remains crucial. As research progresses, the integration of innovative materials, design algorithms, and control frameworks will be essential in driving the development of next-generation systems. By embracing multidisciplinary approaches, future nano-positioning stages can deliver unprecedented levels of accuracy, speed, and reliability to meet the growing demands of nanotechnology, life sciences, and precision manufacturing.

## Figures and Tables

**Table 1:** Performance Comparison of Representative Flexure-Based XY Nanopositioning Stages.

Reference	Parasitic Rotation (µrad)	Resolution (nm)	Cross-Axis Coupling (%)	Natural Frequency (Hz)	Trajectory Tracking Error (nm)	Experimental Stroke (mm)	Size (mm)
[46]	100 (10 mm × 10 mm)	20	0.6	18	2250 (5 mm × 5 mm)	10	385 × 385 (with actuators)
[104]	N.A.	30	N.A.	20.1 (X-axis) 18.4 (Y-axis)	74.9 (1 mm × 0.1 mm)	2	192 × 192 (without actuators)
[105]	N.A.	200	1.3	29.3 (X-axis) 29.6 (Y-axis)	N.A.	11.75 (X-axis) 11.66 (Y-axis)	120 × 120 (without actuators)
[5]	N.A.	106	2.87	4.1	N.A.	54.29	443 × 443 (without actuators)
[106]	N.A.	N.A.	1.0	55.6 (X-axis) 52.6 (Y-axis)	3620 (2 mm × 2 mm)	2	127 × 127 (without actuators)
[78]	N.A.	250	0.79 (X-axis) 0.98 (Y-axis)	43.7 (X-axis) 45.3 (Y-axis)	12300 (0.5 mm × 0.5 mm)	2.13 (X-axis) 2.02 (Y-axis)	280 × 280 (without actuators)
[28]	22.72 (1 mm × 1 mm)	5	0.29 (X-axis) 0.39 (Y-axis)	57.1 (X-axis) 58.9 (Y-axis)	88.30 (1 mm × 1 mm)	2	300 × 300 (with actuators)

**Table 2:** Comparative Analysis of Flexure System Modeling Methods.

Modeling Method	Theoretical Basis	Accuracy	Computational Cost	Primary Application	Key Limitations
PRBM	Rigid-body mechanics with torsional springs	Low to Moderate	Very Low	Conceptual design, initial sizing	Inaccurate for large deformations and complex kinematics
Compliance Matrix/Beam Theory	Continuum mechanics, differential equations	Moderate to High (for simple geometries)	Low	Analytical analysis of basic flexure elements	Becomes intractable for complex 3D systems
Multi-body dynamics	Multi-body dynamics with flexible bodies	High for system-level motion	Moderate	System-level kinematics/dynamics	Contact definition can be complex; depends on input from FEA/analytical models
FEA	Numerical solution of PDEs	Very High	Very High	Detailed design validation, stress and modal analysis	Computationally intensive, not suitable for system-level control

**Table 3:** Comparison of control strategies for nanopositioning systems.

Control Category	Representative Methods	Core Mechanism/Key Feature	Primary Advantages	Primary Limitations/Challenges	Typical Application Scenarios
Active Resonant Control	PPF, PVPF, PAVPF, IRC	Introduces positive feedback to reshape the system’s pole-zero distribution, increasing the damping ratio and suppressing mechanical resonance.	Effectively mitigates resonant peaks. Lays the foundation for high-bandwidth operation. PAVPF enables arbitrary pole placement for 3rd-order models.	Requires accurate system identification. Difficult to extend to higher-order or non-minimum phase systems. Controller complexity scales with the number of modes.	Initial damping of flexure mechanism resonances to improve system stability margins.
Tracking Control	PID, Loop-Shaping (Lead-Lag, Notch), Feedforward	PID provides error-driven regulation. Loop-shaping modifies frequency response. Feedforward improves response speed and reference tracking.	Simple structure, ease of implementation, and reliable performance. Well-established design methodologies. High steady-state accuracy.	Limited capability in handling significant nonlinearities and unmodeled dynamics. Bandwidth is often limited under high-performance demands.	Point-to-point positioning and trajectory tracking for systems with well-behaved dynamics and low uncertainty.
Robust Control	DOB, SMC, $H_{\infty }$, μ-synthesis, ADRC	Designed to maintain stability and performance under model uncertainties and disturbances. DOB/ADRC estimate and compensate disturbances. SMC is insensitive to parameter variations. $H_{\infty }$/μ-synthesis provides frequency-domain guarantees.	Strong robustness against parameter variations, external disturbances, and nonlinearities. Formal guarantees of stability.	Increased design complexity. May lead to conservative performance. Higher computational load (e.g., μ-synthesis).	Applications with significant load variations, unmodeled dynamics, or strong external disturbances.
Adaptive & Learning-Based Control	MRAC, ILC, RC, Neural Networks	Controller parameters adjust online to system changes (Adaptive) or performance improves from operational data (Learning). MRAC uses a reference model. ILC/RC learn from repetitive task errors.	Automatically compensates for time-varying dynamics (e.g., load changes). Achieves extremely high repetitive precision through iteration. Data-driven methods avoid the need for precise models.	Complex design with potential stability risks. Learning controllers require repetitive tasks or large datasets. High computational requirements.	Systems with time-varying parameters or repetitive tasks, e.g., AFM scanning, nano-manufacturing.
Multi-Axis Contour Tracking Control	CCC, TCF, PDC	Focuses on minimizing the coordinated error between axes (contour error) rather than individual axis tracking error. CCC minimizes contour error directly. TCF decouples error dynamics. PDC uses a position-domain approach.	Explicit minimization of contour error improves coordinated motion accuracy. Adaptable to complex contour geometries.	Increased controller complexity. Higher computational demand. Potential stability challenges from multiple interacting control loops.	Applications requiring precise coordinated motion, e.g., micro/nano-fabrication, complex trajectory scanning.

## Data Availability

The original contributions presented in the study are included in the article; further inquiries can be directed to the corresponding author.
